# Between Pleasure and Fragility: Emotional Experience after Chemsex – A Narrative Review

**DOI:** 10.1192/j.eurpsy.2026.12115

**Published:** 2026-07-31

**Authors:** Â. Ferreira, J. Marta, C. Batista, A. F. Reis, T. Cardoso, D. Durães

**Affiliations:** Arrábida’s Local Health Unit, Setúbal, Portugal

## Abstract

**Introduction:**

Chemsex represents a contemporary practice in which the deliberate use of psychoactive substances is combined with sexual activity. Framing this behaviour solely in terms of infectious risk or substance dependence neglects essential dimensions of human experience, such as pleasure, intimacy, the need for social belonging, and emotional wellbeing. To date, limited research has examined how these dimensions are experienced following cessation, when individuals return to sexual activity without chemical enhancement.

**Objectives:**

This narrative review aims to explore how individuals with a history of chemsex experience pleasure, sexuality, and emotional states in the absence of substances, incorporating relevant neurobiological insights.

**Methods:**

A narrative review of the literature indexed in MEDLINE was conducted, with emphasis on high-impact studies concerning chemsex, the neurobiology of pleasure, and mental health outcomes.

**Results:**

Following cessation, many individuals report decreased libido, difficulty achieving orgasm, emotional disconnection, and avoidance of intimacy. Sexual pleasure, previously chemically potentiated, is frequently experienced as blunted or inaccessible. Participants often describe feelings of shame, frustration, loneliness, and, occasionally, grief for previous sexual experiences. Neurobiologically, prolonged substance use induces sustained activation of the nucleus accumbens and the mesolimbic dopaminergic reward system, resulting in a recalibration of baseline pleasure thresholds; consequently, natural rewards may appear less gratifying in the absence of drugs.

**Conclusions:**

The impact of chemsex extends beyond substance consumption, affecting both the capacity to experience pleasure and to form meaningful emotional connections. Emotional blunting following cessation is likely driven by a combination of neurochemical and psychodynamic mechanisms. These findings highlight the necessity of integrated therapeutic approaches that address neurobiological, sexual, and emotional dimensions. Such interventions are essential to support the restoration of pleasure, intimacy, and overall psychological wellbeing, facilitating holistic recovery for individuals with a history of chemsex

**Disclosure of Interest:**

None Declared

